# Occurrence and Levels of Emerging *Alternaria* Mycotoxins Detected in Spices and Herbs Marketed in Italy

**DOI:** 10.3390/toxins17110552

**Published:** 2025-11-05

**Authors:** Katia Gialluisi, Maria Giovanna Nicoletti, Nada El Darra, Michele Solfrizzo, Lucia Gambacorta

**Affiliations:** 1 Institute of Sciences of Food Production (ISPA), National Research Council of Italy (CNR), Via Amendola 122/O, 70126 Bari, Italy; katia.gialluisi@cnr.it (K.G.); michele.solfrizzo@cnr.it (M.S.); 2 Ladisa Srl-Ristorazione, Via Lindemann Z.I, 70132 Bari, Italy; mariagiovannanicoletti@pec.it; 3 Department of Nutrition and Dietetics, Faculty of Health Sciences, Beirut Arab University, Tarik El Jedidah—Beirut, P.O. Box 115020, Riad El Solh, Beirut 1107 2809, Lebanon; n.aldarra@bau.edu.lb

**Keywords:** *Alternaria*, mycotoxins, spices, herbs, mass spectrometry, occurrence, Italy, human exposure

## Abstract

Emerging *Alternaria* mycotoxins tenuazonic acid (TeA), alternariol (AOH), alternariol monomethyl ether (AME), altenuene (ALT) and tentoxin (TEN) were detected in samples of spices and herbs. A total of 92 samples purchased in Italian markets were analyzed by using a UPLC-MS/MS method. TeA was the predominant mycotoxin with the highest percentage of positive samples (53%), followed by AME, AOH and TEN with overall means of 801.5, 2.4, 6.2 and 10.8 µg/kg, respectively. All samples were negative to ALT. The spices with higher levels of mycotoxins were flax seeds, paprika, red chili and licorice; regarding herbs, they were basil, sage and oregano. While TeA was found to be the most abundant mycotoxin equally in Italian and Lebanese samples, AOH and AME showed significantly lower levels in Italian samples, as Lebanese samples contained concentrations up to 14 times higher. Considering the mean levels of TeA in samples, the daily consumption of >8.7 g of flax seeds, >9.3 g of paprika and >5.8 g of red chili will exceed the threshold of toxicological concern (TTC) of TeA, which is 1500 ng/kg bw for a day. The high percentage of positive samples and the high levels of some mycotoxins observed demonstrate the susceptibility of spices and herbs to *Alternaria* mycotoxin contamination. These results provide an overview of emerging *Alternaria* mycotoxins in spices and herbs marketed in Italy and represent a valuable starting point to assess human exposure and support future studies aimed at establishing safe limits to protect human health.

## 1. Introduction

Mycotoxins are toxic secondary metabolites produced by fungi that can contaminate food products, posing significant health risks to humans and animals [1]. Among these, the genus *Alternaria* has garnered attention due to its widespread occurrence and the production of various mycotoxins.

*Alternaria* is a fungal genus that is ubiquitous in the environment and includes saprophytic and pathogenic species [2]. The most common *Alternaria* species include *A. alternata*, *A. tenuissima*, *A. arborescens*, *A. radicina, A. brassicae*, *A. brassicicola*, and *A. infectoria*, which can infest a wide variety of agricultural crops like cereals (wheat, barley, and sorghum), oilseeds, fruits and vegetables, causing considerable losses [3,4]. Particularly, *Alternaria* is a genus well known for its ability to produce a wide spectrum of secondary metabolites, including various phytotoxins related to plant pathogenesis, both host and non-host specific, and mycotoxins that can contaminate food products. Among the many *Alternaria* secondary metabolites, only a few are thought to pose a serious health risk for both humans and animals [5]. These toxins are classified as ‘emerging’ mycotoxins—compounds of potential concern due to their widespread occurrence, high abundance, and possible toxicity—whose health impacts may not yet be fully understood but are recognized for their potential toxicity and prevalence in food supplies [6].

According to the opinion of the EFSA Panel on Contaminants in the Food Chain, alternariol (AOH), alternariol monomethylether (AME), tenuazonic acid (TeA), tentoxin (TEN) and altenuene (ALT) are considered the main *Alternaria* mycotoxins because of their known toxicity and their frequent presence as natural contaminants in food [7].

Several reports have demonstrated genotoxic and mutagenic effects in vitro of AOH and AME, and different studies have indicated that the combination of AOH and AME has additive and synergistic effects on cell viability compared to exposure to each toxin individually [8], while no mutagenic activity has been reported for TEN and TeA [9]. Alternariol was further demonstrated to poison topoisomerases I and II [10], and it is also rapidly absorbed by the human intestinal lumen [11]. Tenuazonic acid is toxic to several animal species, such as mice, chickens, and dogs, and causes esophageal cancer in mice [12] and is the most acutely toxic among the *Alternaria* mycotoxins.

Currently, there is no European legislation setting maximum limits for *Alternaria* mycotoxins in food, but in 2022 the European Commission published Recommendation (EU) 2022/553, which provides guidance for the control of these mycotoxins in certain foods. This Recommendation set indicative levels for alternariol, alternariol monomethyl ether and tenuazonic acid in certain foods, such as processed tomato products, paprika powder, sesame seeds, sunflower oil, tree nuts, dried figs and cereal-based food for infants and young children [13].

Due to this lack of normative regulation, in 2011 the European Food Safety Authority (EFSA) released a scientific report on the potential health risks caused by *Alternaria* mycotoxin contaminations of food and elaborated a dietary exposure assessment. The thresholds of toxicological concern (TTC values) were defined as 2.5 ng/kg body weight per day for the genotoxic compounds AOH and AME and 1500 ng/kg body weight per day for non-genotoxic TEN and TeA [7]. Furthermore, EFSA clearly stated the critical need for more data on the natural occurrence of these mycotoxins in food and feed in order to determine which products are the main contributors to human exposure to *Alternaria* mycotoxins and, therefore, to enable detailed risk assessment.

In the last decade, several papers have been published on the natural occurrence of mycotoxins in herbs and spices due to the current widespread use of these substances worldwide. In Italy there are currently no official data (such as from ISTAT) available on the production or consumption of spices, but the most recent information comes from a private survey conducted by AstraRicerche for Cannamela, a brand of the Montenegro Group and a leading company in the Italian spices and herbs sector. According to this research, the use of spices and herbs in Italy has increased over the past five years: 65.5% of respondents reported using them more frequently than before. The most used spices remain black pepper, saffron, and chili pepper, typical of Mediterranean cuisine.

Unfortunately, spices and herbs are exposed to a wide range of microbial contamination during pre- and post-harvest, despite the low water activity of commodities [14]. Spices are primarily grown in tropical countries, where high temperatures and humidity create favorable conditions for fungal contamination and mycotoxin production [15]. Proper pre- and post-harvest practices are the most effective strategies to mitigate the risk of increased mycotoxin levels [16]. For spices, there are two main types of mycotoxins of concern: AFs and OTA [17], but other mycotoxins such as zearalenone (ZEA), trichothecenes, and fumonisins (FBs) occur as well [18]. Recent studies have investigated mycotoxin contamination in herbs and spices marketed in Italy. Gambacorta et al. (2019) [19] have analyzed the incidence and levels of ALT, AOH, TEN, AME and TeA in samples of spices and herbs marketed in Lebanon, identifying a high contamination, especially by TeA, followed by AME, TEN, AOH and ALT.

Gialluisi et al. (2025) conducted an analysis of 98 samples of spices, herbs, and mixtures marketed in Italy, revealing that mycotoxin occurrence was found in 84% of spices and 60% of herbs, with the occurrence of 1–4 mycotoxins detected in these samples and spices being the more contaminated matrix [18]. This study examined 11 mycotoxins, both regulated and non-regulated, and highlighted the significant prevalence of mycotoxins in the tested samples. The findings underscore the need for ongoing monitoring and risk assessment of mycotoxin contamination in food products. However, this study focused primarily on regulated mycotoxins like aflatoxins and ochratoxin A rather than *Alternaria* toxins specifically. Potortí et al. (2020) investigated 112 samples of spices and aromatic herbs from Italy and Tunisia for mycotoxin contamination, though *Alternaria* toxins were not specifically mentioned [20]. They found varying levels of contamination in coriander, laurel, mint, rosemary, and verbena. The study used HPLC-MS/MS and detected mycotoxins such as AFB2, AFG1, AFG2, T2, and HT2 but found no AFB1 or FB1. Notably, some rosemary and verbena samples showed co-occurrence of two toxins. These findings highlight the need to minimize mycotoxin levels, including *Alternaria* toxins, in agricultural and food production practices to safeguard consumer health.

Furthermore, Lattanzio et al. (2022) developed LC-MS/MS methods for monitoring five *Alternaria* toxins in Italian foods as part of national surveillance programs from 2017 to 2020 [21]. Their focus was primarily on cereals, tomato products, and sunflower seeds, with no specific attention given to spices and herbs. The European Commission has required the collection of occurrence data on *Alternaria* toxins in food and feed since 2012, supported by the EFSA CONTAM Panel’s scientific opinion. While extensive data for cereals and oilseeds indicate significant contamination—especially with tenuazonic acid and tentoxin—there remains a notable gap in occurrence data for spices and herbs. This highlights the urgent need for targeted studies in these categories to ensure comprehensive food safety monitoring.

Although *Alternaria* mycotoxins have been classified as compounds of potential concern due to their widespread occurrence, high abundance, and possible toxicity, research on these toxins remains limited. Additionally, the occurrence of *Alternaria* mycotoxins in spices and herbs has been poorly investigated.

This paper aims to provide an overview of the incidence and levels of emerging *Alternaria* mycotoxins (ALT, AOH, TEN, AME and TeA) in herbs and spices produced worldwide and commercialized in Italy. Using an LC-MS/MS method, the study highlights their risk assessment and, above all, provides occurrence data useful for evaluating human exposure. The findings are particularly important for the European Food Safety Authority (EFSA) as they serve as a preliminary yet significant basis for evaluating human dietary exposure to multiple mycotoxins present in these products. Moreover, the results underscore the urgent need for additional studies to establish safe limits for currently non-regulated mycotoxins. This will inform future risk assessments and regulatory actions aimed at protecting human health.

## 2. Results

### 2.1. Method Performance

The method performance characteristics in terms of mean recoveries and mean repeatability (RSD_r_) reported in Gambacorta et al., 2019 [19], were acceptable for all the analytes tested. In particular, the mean recovery ranged between 74 and 118%, and the mean repeatability ranged between 1 and 7%. For the calculation of the limits of detection (LODs) and quantification (LOQs), the signal-to-noise ratios of 3 and 10, respectively, were used. LOD and LOQ values ranged from 0.82 to 5.36 μg/kg and 2.7–17.86 μg/kg, respectively (Table 1). These values of LODs and LOQs were comparable with those reported by Gambacorta et al., 2019 [19]. An investigation of possible matrix effects on the ionization of *Alternaria* mycotoxins was carried out by comparing calibration curves prepared with standard solutions and matrix-matched calibration curves (7 points) for each mycotoxin. The extracts used for matrix-assisted calibration were prepared from a mixture of three spices (pepper, anise, and nutmeg) and one herb (basil) to closely approximate the highly heterogeneous nature of the samples and to avoid the need for separate calibration curves for each individual matrix. Specifically, it was calculated as the ratio between the slope of the matrix-matched curve and the slope of the calibration curve prepared in the LC mobile phase and multiplied by 100 (%SSE). The results ranged from 74% for TeA to 26% for AME. Signal suppression was observed also for ALT (61%), TEN (40%) and AOH (48%). The lack of a strong purification of sample extracts leads to a suppression of the signal. Anyway, the values of LODs and LOQs were acceptable because of the high performance of the modern/powerful LC-MS/MS apparatus used in this study. In addition, the use of this instrument has allowed us to inject a low amount of matrix equivalent (0.5 mg), obtaining acceptable results. It should be stressed that this method economizes the time and the cost of analysis because it involves sample extraction and partial cleanup before LC-MS/MS determination.

### 2.2. Occurrence of Alternaria Mycotoxins in Different Types of Spices and Herbs

The results of *Alternaria* mycotoxins occurrence are reported in Table 2.

Spices were found to be more susceptible to mycotoxin contamination than herbs. Among the spice samples, TeA was the most prevalent and abundant mycotoxin, with mean concentrations exceeding 1000 µg/kg and the maximum concentration of TeA of 12,611.8 µg/kg; 46% of spice samples tested positive for TeA. AME, AOH, and TEN were present at much lower mean levels (1.4–12.3 μg/kg), with positivity rates ranging from 19% to 28%. ALT was not detected in these samples. In herb samples, TeA was again the most frequently detected toxin (60% positivity), with a mean concentration of 93.1 μg/kg and a maximum of 938.8 μg/kg. AME, AOH and TEN showed lower mean levels (5.0, 8.0 and 4.8 μg/kg, respectively) and moderate detection frequencies (40%, 35% and 20%). ALT was not detected in herbs.

Table 3 shows the mean levels of each *Alternaria* mycotoxin, as well as the sum of the means of the five mycotoxins in species and herbs, classified according to the type of matrix to identify the most contaminated samples. Additionally, the sum of the means for each type of spice/herb was also reported and listed in descending order. Individual mycotoxin data are reported separately in Supplementary Tables S1 and S2. Among the spices, flax seeds, paprika, red chili, licorice and hemp seeds were the most contaminated. The high contamination in these samples was mainly due to TeA, with mean levels from 985.2 to 10,261.9 µg/kg. Among herbs, basil, sage and oregano were the most contaminated samples. In this case as well, TeA was the mycotoxin contributing most to the sum of the means, with average levels ranging from 91.1 to 398.5 µg/kg.

Focusing on spices, TeA accounted for 90.0% to 99.8% of total mycotoxins measured in flax seeds, paprika, red chili, licorice and hemp seeds. Consequently, the combined contribution of AME, AOH, TEN and ALT represented only 0.2 to 10.0% of total contamination. The maximum TeA level (12,611.8 µg/kg) was detected in a paprika sample exceeding the indicative level of 10,000 µg/kg established for paprika by Recommendation (EU) 2022/553. In contrast, TeA, AOH and AME levels in sesame and sunflower seeds did not exceed the indicative values set by the same recommendation.

Anyway, the highest mean level of TEN was observed in cumin seeds (180.5 µg/kg), where TEN contributed 43.7% of the total *Alternaria* mycotoxins. Although lower, the highest average level of AOH was found in licorice (20.1 µg/kg), and the highest average level of AME was detected in coriander (6.1 µg/kg). Regarding herbs, basil and sage were heavily contaminated with TeA, showing mean levels of 398.5 and 111.1 µg/kg, respectively. In basil, TeA was detected together with TEN (13.5 µg/kg), contributing 95.7% of total contamination. In sage, TeA was the major contributor, followed by AME (39.1 µg/kg) and AOH (37.9 µg/kg). In the same basil sample, the highest concentrations of TeA (938.7 µg/kg) and TEN (23.9 µg/kg) were observed, whereas the highest concentrations of AOH (57.9 µg/kg) and AME (65.4 µg/kg) were found in the same sage sample.

**Figure 1:**
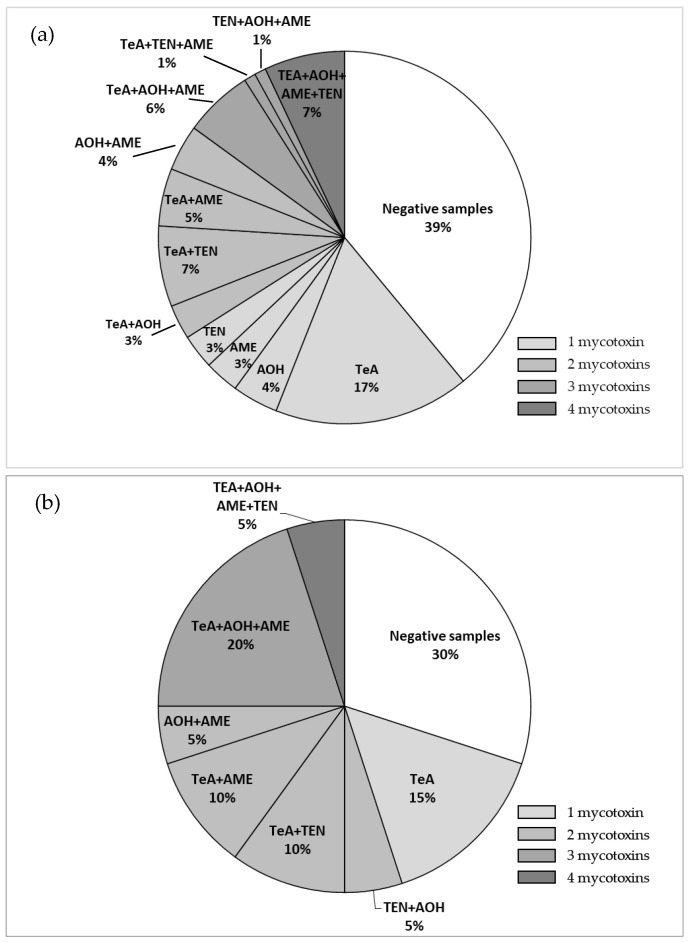
Overview of the co-occurrence of multiple *Alternaria* mycotoxins in (**a**) spices and (**b**) herbs.

An overview of the co-occurrence of multiple *Alternaria* mycotoxins in spices and herbs is shown in Figure 1a,b.

Out of a total of 72 spice samples analyzed, 39% were negative for all five mycotoxins (including white pepper, juniper berries, pumpkin seeds, poppy seeds, anise seeds, fenugreek, and cloves), while 27%, 19%, 8% and 7% of samples contained one, two, three or four mycotoxins, respectively. Among the 20 herb samples, 30% were negative for all five tested mycotoxins, while 15%, 30%, 20% and 5% contained one, two, three or four mycotoxins, respectively. No spice and herb samples contained all five mycotoxins. In addition, Figure 1a,b showed the different combinations of mycotoxins observed in spices and herbs.

In Table 4, spice samples were classified according to the edible part of the plants and listed from the highest to the lowest sum of mean total mycotoxin levels. For herb samples, which all originated from the same plant part (leaf), no classification was applied. The highest total mycotoxin levels were observed in fruits (red chili and paprika) with a contamination level of 7711.0 µg/kg, followed by seeds (15 different types), roots (licorice, ginger, and turmeric), bark (cinnamon), berries (6 different types), bulbs (onion and garlic) and buds (cloves). The highest mean levels of TeA, TEN and AME were found in fruits (7668.2, 27.3, and 4.1 µg/kg, respectively), whereas the highest mean level of AOH was detected in bark (13.6 µg/kg). Notably, the two gem samples were completely free of *Alternaria* contamination.

## 3. Discussion

### 3.1. Comparison of Spices and Herbs Marketed in Italy and Lebanon

**Figure 2:**
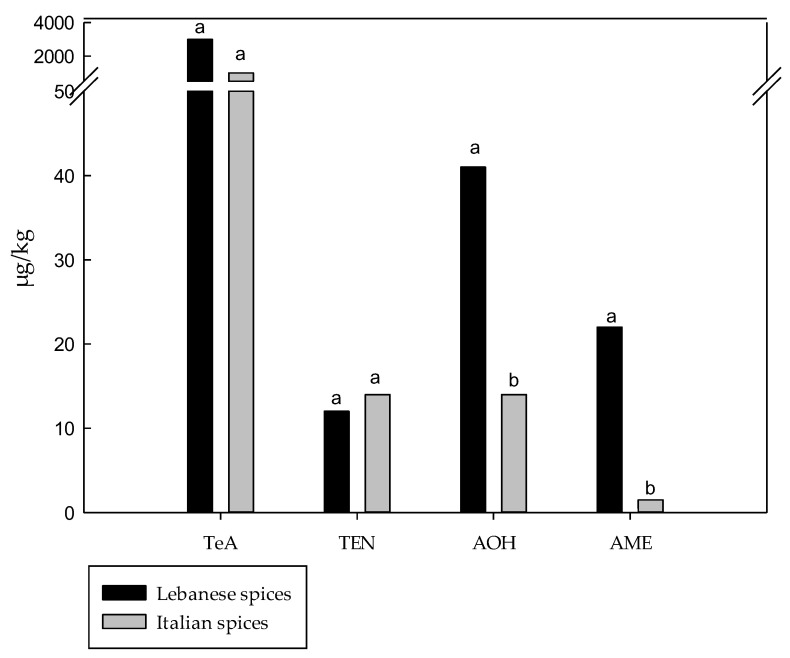
Comparison of *Alternaria* mycotoxin mean levels in Lebanese spices (*n* = 57) and Italian spices (*n* = 54). Different letters indicate significant differences between means (*p* < 0.05); means followed by different letters (a,b) differ significantly according to the unpaired *t*-test with Welch correction (one-tailed *p*-value).

**Figure 3:**
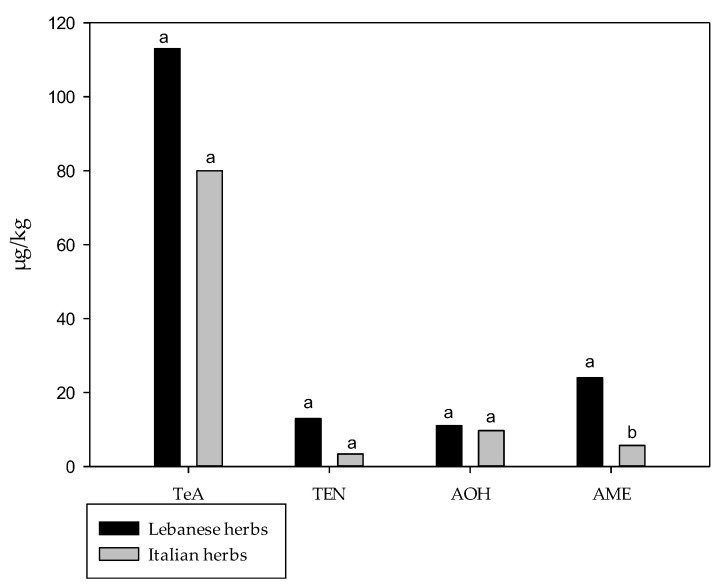
Comparison of *Alternaria* mycotoxin mean levels in Lebanese herbs (*n* = 19) and Italian herbs (*n* = 17). Different letters indicate significant differences between means (*p* < 0.05); means followed by different letters (a,b) differ significantly according to the unpaired *t*-test with Welch correction (one-tailed *p*-value).

The results obtained in this study were compared with those reported by Gambacorta et al., 2019 [19] that evaluated the presence of TeA, AOH, AME, TEN and ALT in spices and herbs marketed in Lebanon. Both studies highlighted a significant presence of *Alternaria* mycotoxins in spices and herbs, likely due to the absence of specific international regulations for these mycotoxins in such foodstuffs. In Figure 2 the average levels of each *Alternaria* mycotoxin in Italian and Lebanese spices are compared. A total of 18 different spice types were considered, corresponding to the types common to both Italy and Lebanon: paprika, red chili, cumin, coriander seeds, nutmeg, cinnamon, turmeric, garlic, sesame, anise, ginger, black pepper, onion, fennel, cardamom seeds, white pepper, fenugreek and cloves. Similarly, Figure 3 compares the mean levels of the five mycotoxins in Italian and Lebanese herbs, considering eight herb types common to both countries: basil, sage, oregano, mint, parsley, thyme, rosemary and marjoram.

Significantly higher mean levels of AOH and AME were measured in Lebanese spices compared to Italian spices, whereas no significant differences were observed for TeA and TEN. The mean levels of AOH and AME in Lebanese spices were approximately 9 and 14 times higher than those observed in the Italian samples, respectively. In particular, AOH and AME in Lebanese spices were 41.3 and 22.5 µg/kg, and AOH and AME in Italian spices were 4.6 and 1.6 µg/kg, respectively. Regarding herbs, significant differences were observed only for AME, with Lebanese samples showing higher levels than Italian herbs (25.8 and 5.8 µg/kg, respectively), while no differences were noted for TeA, TEN and AOH. Since ALT was not detected in any Italian spices and herbs samples, no statistical analysis was performed for this mycotoxin. TeA was the predominant mycotoxin in both Italian and Lebanese spices, with mean levels of 812.1 and 2954.6 µg/kg, respectively, though the difference was not significant. Similarly, in herbs, TeA exhibited the highest mean levels in both Italy and Lebanon (81.1 µg/kg and 113.4 µg/kg, respectively).

In Italy, paprika and red chili were the most heavily contaminated spices, showing high mean mycotoxin levels. Similarly, the study by Gambacorta et al. (2019) [19] confirmed high contamination of the same spices in Lebanon. Notably, total mycotoxin concentration in Lebanese paprika reached 24,463.3 µg/kg, over 2.5 times higher than the 9698.0 µg/kg detected in Italian samples. Among individual toxins, TeA was the most abundant in both, with 24,337.3 µg/kg in the Lebanese paprika versus 9640.6 µg/kg in the Italian. TEN and AOH were also found at higher levels in the Lebanese samples (71.8 µg/kg and 28.7 µg/kg, respectively) compared to the Italian (36.6 µg/kg and 13.0 µg/kg), while AME showed a nearly six-fold increase in the Lebanese samples (29.2 µg/kg) compared to the Italian one (5.1 µg/kg). Similarly, total mycotoxin concentration in Lebanese red chili reached 27,323 µg/kg, more than six times higher than the 4297.6 µg/kg detected in Italian samples. In both cases, TeA was the dominant toxin, with an exceptionally high level of 27,255.5 µg/kg in the Lebanese samples compared to 4274.9 µg/kg in the Italian one. Other mycotoxins such as TEN, AOH, and AME were also found in higher concentrations in the Lebanese samples. Regarding herb samples, analysis of basil revealed a notable difference in *Alternaria* mycotoxin contamination between Italian and Lebanese herbs. In the Italian basil, two *Alternaria* mycotoxins were detected: TeA at 398.5 µg/kg and TEN at 13.5 µg/kg. In contrast, no *Alternaria* mycotoxins were detected in the Lebanese basil samples, suggesting a cleaner mycotoxin profile possibly due to different environmental conditions, agricultural practices, or post-harvest handling. Sage samples showed moderate contamination in both countries, with slightly higher total mycotoxins in Lebanese sage (253.4 µg/kg) compared to Italian (192.4 µg/kg). In both cases, TeA was detected at comparable levels: 111.1 µg/kg in the Italian samples and 137.6 µg/kg in the Lebanese. AOH and AME were also present in both samples, with AME being notably higher in the Lebanese sage (80.3 µg/kg) compared to the Italian sage (39.1 µg/kg). TEN was detected only in the Lebanese sage, while ALT was not detected in either. Overall, these findings suggest that Lebanese spices, and to a lesser extent herbs, were more heavily contaminated with *Alternaria* mycotoxins than Italian products. These differences may reflect variations in climatic conditions, agricultural practices and post-harvest handling. The results underscore the importance of region-specific monitoring and control measures, particularly in countries where environmental or processing conditions may promote higher mycotoxin contamination [18].

### 3.2. Comparison with the Scientific Literature Data

The high susceptibility of spices and herbs to contamination by *Alternaria* mycotoxins observed in this study was consistent with the findings of previous investigations. Notably, the pronounced contamination levels detected in paprika agreed with those reported in a study conducted in Italy [22]. In that study, paprika exhibited the highest concentrations of *Alternaria* toxins among the spices analyzed, with TEA reaching 8248.5 µg/kg, AOH 428.4 µg/kg and ALT 40.3 µg/kg. Other studies in the literature investigated the occurrence of *Alternaria* toxins in paprika. The high levels of TeA detected in all paprika samples analyzed in the present study (ranging from 7602.3 µg/kg to 12,611.8 µg/kg) were comparable to the 8800 µg/kg of TeA reported by Arcella et al. (2016) [3], but higher than the 2900 µg/kg reported by Asam et al. (2012) [23]. In addition, in a study by Mujahid et al., 2020 [24], only one paprika powder sample (TeA 7356 µg/kg) out of eight analyzed was found below the indicative level, while the remaining samples exceeded this threshold, with concentrations ranging from 10,163 to 18,856 µg/kg. These values were higher than those detected in our study. Similarly, in the study conducted by da Cruz Cabral et al., 2016, TeA was the most prevalent mycotoxin in moldy peppers, occurring in 50% of samples at concentrations ranging from 8 to 11,422 µg/kg. AOH and AME were detected less frequently (21% and 29%, respectively) and at lower levels (3–98 µg/kg and 7–272 µg/kg, respectively) [25]. The absence of *Alternaria* mycotoxins in cloves observed in our study could be due to their essential oils that were highly effective against molds, as indicated in a study by Hashem M. et al. (2010) [26]. It should be underlined that all samples of the examined spices and herbs in the present study appeared healthy and devoid of any mold symptom. In Mujahid et al., 2020 [24], TeA was detected in nearly all herb samples, reaching levels up to 748 µg/kg, which were lower than the 938.8 µg/kg observed in the present study. Other *Alternaria* toxins were found at concentrations ranging from <10 to 113 µg/kg in Mujahid et al., 2020, compared with 0.4–65.4 µg/kg in our study. ALT was not detected in both studies. Table 5 compares the maximum concentrations of the predominant mycotoxin (TeA) in paprika and herbs between the present study and previously published data.

Consumers’ exposure to *Alternaria* mycotoxins could be caused by contaminated spices and herbs. Unluckily, there were no data available on the intake of spices and herbs in Italy when the results reported herein were analyzed, but there were data on the intake of spices and herbs in southern India (10–29 g/person/day), Norway (2.7 g/person/day) and Thailand (4.9–26.1 g/person/day) [27]. Using the TTC for TeA (1500 ng/kg bw/day) [7] and the mean mycotoxin levels measured in this study, safe daily consumption was calculated according to the formula described by Gambacorta et al., 2019 [19]. The results suggested that intake above 8.7 g of flax seeds, 9.3 g of paprika, and 21.0 g of red chili would exceed the TTC.

## 4. Conclusions

Spices and herbs can be regarded as important vectors for various microorganisms, determining possible health problems for the consumer and quality and shelf-life problems for foods. By virtue of the results presented in this study, spices and herbs clearly demonstrated a high susceptibility to the emerging *Alternaria* mycotoxins. In fact, out of a total of 92 samples of spices and herbs, a high percentage of these (66%) were contaminated by 1–4 mycotoxins. Flax seeds, paprika, red chili, basil, and sage presented the highest contaminations in *Alternaria* mycotoxins. TeA was the predominant mycotoxin, followed by AME, AOH and TEN, whereas all samples were negative to ALT. Thus, our findings demonstrate the need for a worldwide regulation for *Alternaria* mycotoxins and, above all, the need to implement guidelines concerning the phases of storage, processing, packaging and distribution to identify critical points that promote further growth of *Alternaria* species and so a greater accumulation of mycotoxins in this foodstuff.

## 5. Materials and Methods

### 5.1. Sampling

Ninety-two samples of different kinds of herbs and spices were randomly collected from different markets in Bari. Among these, 72 samples were spices (67 single spices and 5 mixtures of spices) and 20 samples were herbs (19 single herbs and 1 mixture of herbs). A detailed description of the samples was given in Table 6. The samples in the form of berries (pepper and juniper berries), seeds (sunflower, flax, sesame, cardamom, poppy, chia, pumpkin, fennel, hemp, and mustard), and sticks (cinnamon) were carefully ground using a blade mill, thus obtaining homogeneous powdered matrices. All other samples were purchased already ground. Each sample was transported to CNR-ISPA (Bari) and stored at room temperature, then cataloged and numbered with an identification code before being analyzed by UPLC-MS/MS for the simultaneous determination of ALT, AOH, TEN, AME and TeA.

### 5.2. Chemicals and Reagents

Standard solutions for each mycotoxin were purchased from Romer Labs Diagnostic (Tullin, Austria). Specifically, separated standard solutions of ALT (1 μg/mL), AOH (1 μg/mL), TEN (1 μg/mL), AME (1 μg/mL) and TeA (1 μg/mL) in acetonitrile (ACN) were prepared. Chromatography-grade methanol (MeOH), ACN, formic acid and bicarbonate of ammonium were obtained from Sigma-Aldrich (Milan, Italy). Regenerated cellulose filters (0.45 μm) were acquired by Sartorius Stedim Biotech (Goettingen, Germany). All the above-mentioned reference substances were gifted with an analytical quality certificate. Ultrapure water was produced by using a Milli-Q system (Millipore, Bedford, MA, USA).

### 5.3. Determination of Alternaria Mycotoxins

#### 5.3.1. Mycotoxins Extraction

To determine ALT, AOH, TEN, AME and TeA in spice and herb samples, the innovative method performed by Gambacorta et al., 2019 [19] was used and is described below. In detail, 5 g of ground spice or herb were transferred to a 250 mL Pyrex screw-capped glass flask and thoroughly extracted with 20 mL of acetonitrile: H_2_O/formic acid mixture (49:50:1, *v/v/v*). For some samples of herbs rich in fiber, it was necessary to add a higher volume, up to a maximum of 60 mL, because of their high water absorption capacity. The final analyte concentrations were calculated on a per mass basis (µg/kg dry weight) after correcting for the actual extraction volume and any dilution factors, ensuring data comparability across samples. Then, each individual sample was first shaken manually for 10 s to obtain a homogeneous suspension and then sonicated for 30 min. After centrifugation (4000× *g*, 3 min), 200 μL of extract were diluted with 800 μL of ultrapure H_2_O, manually shaken and left overnight at 4 °C. Following this, the extract was filtered with a regenerated cellulose filter (0.45 μm) syringe and analyzed by UPLC-MS/MS.

Three spices (pepper, anise, and nutmeg) and an herb (basil) were mixed, ground and used to prepare the extracts for matrix-assisted calibration solutions. Standard solutions of TeA, AOH, ALT, TEN and AME were diluted with acetonitrile and used to prepare two mixed spiking solutions. Appropriate amounts of these solutions were added to the extracts for matrix-assisted calibration solutions to have seven mixed calibration solutions at different increasing concentrations. For the analytical procedure, the same recovery experiments carried out by Gambacorta et al., 2019 [19] were considered. Mean recoveries ranged from 74% for AME to 118% for ALT. The limits of detection (LOD) and quantification (LOQ) of each mycotoxin were calculated as 3 times and 10 times the noise, respectively.

#### 5.3.2. LC-MS/MS Equipment and Parameters

A triple quadrupole API 5000 system (Applied Biosystems, Foster City, CA, USA), equipped with an ESI interface and an Acquity UPLC system comprising a binary pump and a micro autosampler from Waters (Milford, MA, USA), was used for LC-MS/MS analyses. Optimized instrument parameters were taken from Gambacorta et al., 2019 [19]. In detail, interface conditions were TEM, 450 C; CUR, nitrogen, 20 psi; GS1, air, 50 psi; GS2, air, 30 psi; and ion spray voltage, −4500 V. For the separation of *Alternaria* mycotoxins, a C_18_ Gemini analytical column (2 mm × 150 mm, 5 μm particles; Phenomenex Inc. Torrance, CA, USA) was used, and the column oven was set at 40 °C. The flow rate of the mobile phase was 300 μL/min, and the injection volume was 10 μL. The mobile phase consisted of a binary linear gradient of a mixture of (A), H_2_O:MeOH (95:5, *v/v*) 1 mM ammonium bicarbonate, and (B), MeOH used as follows: 100% A for 1 min, then brought to 100% B in 10 min, then maintained at 100% B for 8 min, then brought to 100% A in 0.5 min and left to equilibrate for 4.5 min before the next run. The total run, for each sample, lasted 24 min. For LC-MS/MS analyses, the ESI interface was used in negative ion mode. The mass spectrometer operated in MRM (multiple reaction monitoring) mode. The optimized MS/MS conditions for *Alternaria* mycotoxins are listed in Table 7: in particular, four transitions for confirmation and one transition for quantification for all mycotoxins. Quantification of ALT, TeA, TEN, AME and AOH in 92 dried and ground samples of spices and herbs was performed by measuring peak areas in the MRM chromatogram and comparing them with the relevant matrix-matched calibration curves. The calibration ranges in the matrix ranged between 0.005 and 0.4 ng injected for ALT, TeA and TEN and 0.0025 and 0.8 ng injected for AME and AOH. For matrix calibration, the amounts of toxin injected refer to 0.0005 g of matrix injected. Therefore, the final concentrations ranged between 10 and 800 ng/g for ALT, TeA and TEN; 5 and 40 ng/g for AME; and 20 and 1600 ng/g for AOH. The identity of each analyte in the chromatograms of the sample extracts was further confirmed by comparing the retention times and the ion ratios with the calibration standards (Commission Decision 2002/657/EC) [28].

#### 5.3.3. Evaluation of Results

Not detected (ND) results were treated as LOD/2, and those < LOQ but >LOD as 1/2 LOQ, according to the middle-bound approach reported by (EFSA, 2010) [12].

### 5.4. Statistical Analysis

MS Excel 2013 software (Microsoft Corporation, Redmond, WA, USA) was used to calculate mean, weighted mean and median. Statistical analyses were performed by using the GraphPad Instat 3.10 software (Instat, San Diego, CA, USA). Data represented in Figure 2 and Figure 3 were subjected to the unpaired t-test with Welch correction (one-tailed *p*-value).

## Figures and Tables

**Table 1:** Results of the limits of detection (LODs) and quantification (LOQs) in this study in a mixture of spices and herbs.

*Alternaria* Mycotoxins	LOD (µg/kg)	LOQ (µg/kg)
TeA	1.0	3.4
AOH	2.2	7.4
AME	0.8	2.7
TEN	3.2	11.2
ALT	5.4	17.9

**Table 2:** Summary of the results obtained for the occurrence of *Alternaria* mycotoxins (TeA, AME, AOH, TEN, ALT) in (**a**) 72 spice samples and (**b**) 20 herb samples commercialized in Italy.

**(** **a)**	**Spices** **(*n* = 72)**	**TeA**	**AME**	**AOH**	**TEN**	**ALT**
	Mean (µg/kg)	1040.3	1.4	5.0	12.3	nd
	Median (µg/kg)	0.5	0.4	1.1	1.6	nd
	Max (µg/kg)	12,611.8	16.9	35.6	359.5	nd
	n. positives	33	20	18	14	0
	% positives	46	28	25	19	0
**(** **b)**	**Herbs** **(*n* = 20)**	**TeA**	**AME**	**AOH**	**TEN**	**ALT**
	Mean (µg/kg)	93.1	5.0	8.0	4.8	nd
	Median (µg/kg)	25.7	0.4	1.1	1.6	nd
	Max (µg/kg)	938.8	65.4	57.9	23.9	nd
	n. positives	12	8	7	4	0
	% positives	60	40	35	20	0

nd: not detected.

**Table 3:** Mean levels of *Alternaria* mycotoxins in different types of (**a**) spices and (**b**) herbs.

**(a)**	**Spices (72)**	**TeA** **μg/kg (n.pos)**	**TEN** **μg/kg (n.pos)**	**AOH μg/kg (n.pos)**	**AME** **μg/kg (n.pos)**	**ALT** **μg/kg (n.pos)**	**Sum of Means** **μg/kg**
	Flax seeds (2)	10,261.9 (2)	17.8 (1)	1.1 (0)	0.4 (0)	2.7 (0)	10,283.9
	Paprika (3)	9640.6 (3)	36.6 (3)	13.0 (2)	5.1 (3)	2.7 (0)	9698.0
	Red chili (4)	4274.9 (4)	14.0 (1)	3.6 (1)	2.4 (3)	2.7 (0)	4297.6
	Licorice (1)	1158.3 (1)	53.6 (1)	20.1 (1)	3.8 (1)	2.7 (0)	1238.5
	Hemp seeds (1)	985.2 (1)	105.6 (1)	1.1 (0)	0.4 (0)	2.7 (0)	1095.0
	Mix spices (5)	841.0 (2)	7.2 (2)	1.1 (0)	0.4 (0)	2.7 (0)	852.4
	Cumin (2)	228.6 (2)	180.5 (2)	1.1 (0)	0.4 (0)	2.7 (0)	413.3
	Sichuan pepper (1)	251.4 (1)	1.6 (0)	18.5 (1)	3.4 (1)	2.7 (0)	277.6
	Coriander seeds (1)	227.4 (1)	1.6 (0)	15.3 (1)	6.1 (1)	2.7 (0)	253.1
	Mustard seeds (1)	104.9 (1)	28.7 (1)	1.1 (0)	0.4 (0)	2.7 (0)	137.8
	Nutmeg (3)	109.0 (2)	1.6 (0)	1.1 (0)	0.4 (0)	2.7 (0)	114.8
	Sunflower seeds (1)	59.5 (1)	1.6 (0)	1.1 (0)	0.4 (0)	2.7 (0)	65.3
	Cinnamon (5)	36.3 (2)	1.6 (0)	13.6 (4)	1.6 (2)	2.7 (0)	55.8
	Chia seeds (1)	37.0 (1)	1.6 (0)	12.4 (1)	0.4 (0)	2.7 (0)	54.1
	Turmeric (3)	39.1 (2)	1.6 (0)	1.1 (0)	0.4 (0)	2.7 (0)	44.9
	Garlic (7)	16.7 (3)	3.0 (1)	10.0 (3)	3.2 (4)	2.7 (0)	35.6
	Sesame seeds (2)	17.2 (2)	1.6 (0)	5.9 (1)	3.8 (1)	2.7 (0)	31.2
	Ginger (3)	20.7 (1)	1.6 (0)	1.1 (0)	0.4 (0)	2.7 (0)	26.5
	Black pepper (5)	4.4 (1)	1.6 (0)	6.1 (2)	0.8 (2)	2.7 (0)	15.6
	Onion (5)	0.5 (0)	2.4 (1)	4.5 (1)	1.3 (1)	2.7 (0)	11.4
	Fennel seeds (3)	0.5 (0)	2.9 (1)	1.1 (0)	0.4 (0)	2.7 (0)	7.6
	Cardamon seeds (1)	0.5 (0)	2.9 (1)	1.1 (0)	0.4 (0)	2.7 (0)	7.6
	Green pepper (2)	0.5 (0)	1.6 (0)	1.1 (0)	0.8 (1)	2.7 (0)	6.7
	White pepper (3)	0.5 (0)	1.6 (0)	1.1 (0)	0.4 (0)	2.7 (0)	6.3
	Juniper berries (1)	0.5 (0)	1.6 (0)	1.1 (0)	0.4 (0)	2.7 (0)	6.3
	Pumpkin seeds (1)	0.5 (0)	1.6 (0)	1.1 (0)	0.4 (0)	2.7 (0)	6.3
	Poppy seeds (1)	0.5 (0)	1.6 (0)	1.1 (0)	0.4 (0)	2.7 (0)	6.3
	Anice seeds (1)	0.5 (0)	1.6 (0)	1.1 (0)	0.4 (0)	2.7 (0)	6.3
	Fenugreek (1)	0.5 (0)	1.6 (0)	1.1 (0)	0.4 (0)	2.7 (0)	6.3
	Cloves (2)	0.5 (0)	1.6 (0)	1.1 (0)	0.4 (0)	2.7 (0)	6.3
**(b)**	**Herbs (20)**	**TeA** **μg/kg** **(n.pos)**	**TEN** **μg/kg** **(n.pos)**	**AOH** **μg/kg (n.pos)**	**AME** **μg/kg** **(n.pos)**	**ALT** **μg/kg (n.pos)**	**Sum of** **Means** **μg/kg**
	Basil (3)	398.5 (2)	13.5 (2)	1.1 (0)	0.4 (0)	2.7 (0)	416.2
	Sage (2)	111.1 (2)	1.6 (0)	37.9 (2)	39.1 (2)	2.7 (0)	192.4
	Oregano (3)	91.1 (3)	5.5 (1)	8.2 (1)	2.7 (2)	2.7 (0)	110.2
	Mix herbs (1)	51.9 (1)	1.6 (0)	13.5 (1)	5.0 (1)	2.7 (0)	74.7
	Mint (1)	19.7 (1)	1.6 (0)	10.9 (1)	1.3 (1)	2.7 (0)	36.2
	Chives (1)	0.5 (0)	17.5 (1)	10.7 (1)	0.4 (0)	2.7 (0)	31.8
	Parsley (3)	16.7 (1)	1.6 (0)	1.1 (0)	0.4 (0)	2.7 (0)	22.5
	Dill (1)	14.9 (1)	1.6 (0)	1.1 (0)	0.4 (0)	2.7 (0)	20.7
	Thyme (1)	0.5 (0)	1.6 (0)	12.4 (1)	1.3 (1)	2.7 (0)	18.5
	Rosemary (3)	10.9 (1)	1.6 (0)	1.1 (0)	0.7 (1)	2.7 (0)	17.0
	Marjoram (1)	0.5 (0)	1.6 (0)	1.1 (0)	0.4 (0)	2.7 (0)	6.3

**Table 4:** Weighted means of *Alternaria* mycotoxins in spices classified according to the edible part of plant.

SPICES							
	Fruits	Seeds	Roots	Bark	Berries	Bulbs	Buds
	red chili (3)	chia (1)	licorice (1)	cinnamon (5)	green pepper (2)	onion (5)	cloves (2)
	paprika (3)	sunflower (1)	ginger (3)		black pepper (5)	garlic (7)	
		pumpkin (1)	turmeric (3)		pink pepper (1)		
		mustard (1)			white pepper (3)		
		sesame (2)			Sichuan pepper (1)		
		flax (2)			juniper (1)		
		anise (1)					
		fennel (3)					
		cardamon (1)					
		poppy (1)					
		hemp (1)					
		nutmeg (3)					
		cumin (2)					
		coriander (1)					
		fenugreek (1)					
N. of samples	6	22	7	5	13	12	2
Mycotoxins	Weighted mean of means (μg/kg)						
TeA	7668.2	1034.6	191.1	36.3	22.2	9.9	0.5
AOH	8.7	2.7	3.9	13.6	4.4	7.5	1.1
AME	4.1	1.0	0.9	1.6	0.9	2.4	0.4
TEN	27.3	25.5	9.0	1.6	1.6	2.7	1.6
ALT	2.7	2.7	2.7	2.7	2.7	2.7	2.7
Sum	7711.0	1066.4	207.6	55.8	31.7	25.3	6.3

**Table 5:** Comparison of maximum TeA level in paprika and herbs with the scientific literature.

Study	Matrix	Max TeA Level (µg/kg)
Present study	Paprika	12,611.8
Arcella et al., 2016 [3]	Paprika	8800
Asam et al., 2012 [23]	Paprika	2900
Mujahid et al., 2020 [24]	Paprika	18,856
da Cruz Cabral et al., 2016 [25]	Peppers	11,422
Present study	Herbs	938.8
Mujahid et al., 2020 [24]	Herbs	748

**Table 6:** List of spice and herb samples commercialized in Italy.

Spices (67)	Mix Spices (5)	Herbs (19)	Mix Herbs (1)
Pink pepper (1)	Mix pepper ^1^ (1)	Chive (1)	Mix herbs ^5^ (1)
Green pepper (2)	Mix Berberè ^2^ (1)	Dill (1)	
Black pepper (5)	Curry ^3^ (2)	Sage (2)	
White pepper (3)	Mix Creola ^4^ (1)	Basil (3)	
Sichuan pepper (1)		Parsley (3)	
Juniper berries (1)		Marjoram (1)	
Sunflower seeds (1)		Rosmary (3)	
Pumpkin seeds (1)		Thyme (1)	
Mustard seeds (1)		Oregano (3)	
Sesame seeds (2)		Mint (1)	
Flax seeds (2)			
Anise seeds (1)			
Fennel seeds (3)			
Chia seeds (1)			
Cardamom seeds (1)			
Poppy seeds (1)			
Hemp seeds (1)			
Cumin (2)			
Coriander (1)			
Licorice (1)			
Fenugreek (1)			
Paprika (3)			
Red chili (3)			
Nutmegs (3)			
Cinnamon (5)			
Ginger (3)			
Curcuma (3)			
Cloves (2)			
Onion (5)			
Garlic (7)			

^1^ Mix pepper: black pepper, pink pepper, white pepper, allspice, Sichuan pepper; ^2^ Mix Berberè: sweet paprika, chili pepper, cardamom, coriander, fenugreek, allspice, cloves, black pepper, ginger, cinnamon; ^3^ Curry: black pepper, cumin, coriander, turmeric, cloves, ginger, chili pepper, cardamom, garlic, allspice, fennel seeds, white pepper; ^4^ Mix Creola: white pepper, black pepper, green pepper, Schinus berries, allspice; ^5^ Mix herbs: rosemary, marjoram, sage, thyme, bay leaf, lavender.

**Table 7:** MS/MS parameters for mycotoxin detection by the multiple reaction monitoring (MRM) method.

Analyte	Precursor Ion	Q1 (*m/z*)	Q3 (*m/z*)	DP (V)	EP (V)	CE(V)	CXP (V)
TeA	[TeA-H]^−^	196.2	111.7	−100	−4	−34	−10
			139.1			−28	
			69.0 *			−62	
			82.8			−58	
AOH	[AOH-H]^−^	257.2	215.0	−150	−3	−35	−15
			147.1			−44	
			185.2 *			−38	
			156.9			−40	
AME	[AME-H]^−^	271.4	256.0	−120	−10	−30	−18
			227.9			−40	−16
			213.2			−50	−15
			183.0 *			−55	−12
TEN	[TEN-H]^−^	413.5	141.1	−150	−5	−30	−15
			213.8 *	−150		−35	
			271.2	−160		−23	
			339.2	−160		−39	
ALT	[ALT-H]^−^	291.4	202.9	−160	−5	−45	−15
			248.0			−35	
			160.9 *			−58	
			188.8			−46	

* Transition used for quantitation. Q1: first quadrupole; Q3: third quadrupole; DP: declustering potential; EP: entrance potential; CE: collision energy; CXP: collision cell exit potential.

## Data Availability

The original contributions presented in this study are included in the article. Further inquiries can be directed to the corresponding author.

## Supplementary Materials

The following supporting information can be downloaded at https://www.mdpi.com/article/10.3390/toxins17110552/s1, Table S1: Level (µg/kg) of *Alternaria* mycotoxins in 72 spice samples without application of the middle-bound approach. Table S2: Level (µg/kg) of *Alternaria* mycotoxins in 20 herb samples without application of the middle-bound approach.
