# Supplementary material for: Occurrence and Levels of Emerging Alternaria Mycotoxins Detected in Spices and Herbs Marketed in Italy
Source: Toxins (Basel). 2025 Nov 5;17(11):552. doi: 10.3390/toxins17110552 (PMC12656289; doi:10.3390/toxins17110552)
Supplement: Supplementary file 1 [file toxins-17-00552-s001.zip › toxins-3945372-supplementary.pdf]

Table S1. Level (µg/kg) of *Alternaria* mycotoxins in 72 spices samples without application of the middle-bound approach.

Spices (*n*=72)	(µg/kg)				
	**TeA**	**TEN**	**AOH**	**AME**	**ALT**
Pink pepper	12.640	nd	nd	nd	nd
Long black pepper	nd	nd	10.143	nd	nd
Black pepper_a	20.138	nd	nd	nd	nd
Black pepper_b	nd	nd	nd	nd	nd
Whole black pepper	nd	nd	17.099	<LOQ	nd
Ground black pepper	nd	nd	nd	<LOQ	nd
Sichuan pepper	251.416	nd	18.546	3.381	nd
Green pepper_a	nd	nd	nd	<LOQ	nd
Green pepper_b	nd	nd	nd	nd	nd
White pepper_a	nd	nd	nd	nd	nd
White pepper_b	nd	nd	nd	nd	nd
White pepper_c	nd	nd	nd	nd	nd
Chia seeds	37.041	nd	12.360	nd	nd
Hulled sunflower seeds	59.472	nd	nd	nd	nd
Yellow mustard seeds	104.975	28.707	nd	nd	nd
Pumpkin seeds	nd	nd	nd	nd	nd
Black sesame seeds	15.956	nd	nd	nd	nd
Sesame seeds	18.504	nd	10.627	7.124	nd
Yellow flax seeds_a	8707.813	33.902	nd	nd	nd
Yellow flax seeds_b	11,816.071	nd	nd	nd	nd
Hemp seeds	985.223	105.573	nd	nd	nd
Fennel seeds_a	nd	<LOQ	nd	nd	nd
Fennel seeds_b	nd	nd	nd	nd	nd
Fennel seeds_c	nd	nd	nd	nd	nd
Anise seeds	nd	nd	nd	nd	nd
Poppy seeds	nd	nd	nd	nd	nd
Cardamom seeds	nd	nd	nd	nd	nd
Licorice	1158.260	53.584	20.112	3.818	nd
Juniper berries	nd	nd	nd	nd	nd
Cloves_a	nd	nd	nd	nd	nd
Cloves_b	nd	nd	nd	nd	nd
Fenugreek	nd	nd	nd	nd	nd
Freeze-dried garlic	nd	nd	10.995	<LOQ	nd
Garlic_a	nd	nd	nd	nd	nd
Garlic_b	nd	nd	nd	nd	nd
Granulated garlic_a	42.634	11.200	35.635	16.863	nd
Granulated garlic_b	34.051	nd	nd	<LOQ	nd
Granulated garlic_c	nd	nd	16.669	nd	nd
Ground garlic	38.199	nd	nd	<LOQ	nd
Paprika	8707.821	33.921	nd	<LOQ	nd
Hot paprika_a	7602.304	45.689	14.640	8.931	nd
Hot paprika_b	12,611.750	30.183	23.337	5.094	nd
Chili powder	11,816.087	51.052	11.027	5.318	nd
Chili powder	4654.258	nd	nd	<LOQ	nd
Whole chili	616.717	nd	nd	2.710	nd

Ground cinnamon	145.474	nd	18.570	<LOQ	nd
Cinnamon sticks	34.487	nd	16.821	nd	nd
Cinnamon_a	nd	nd	14.204	5.606	nd
Cinnamon_b	nd	nd	17.174	nd	nd
Cinnamon_c	nd	nd	nd	nd	nd
Ginger_a	61.268	nd	nd	nd	nd
Ginger_b	nd	nd	nd	nd	nd
Ginger_c	nd	nd	nd	nd	nd
Cumin_a	254.620	nd	nd	nd	nd
Cumin_b	202.649	359.474	nd	nd	nd
Turmeric_a	37.041	nd	nd	nd	nd
Turmeric_b	79.704	nd	nd	nd	nd
Turmeric_c	nd	nd	nd	nd	nd
Coriander	227.383	nd	15.334	6.137	nd
Ground nutmeg	256.222	nd	nd	nd	nd
Nutmeg powder	70.237	nd	nd	nd	nd
Nutmeg	nd	nd	nd	nd	nd
Onion flakes	nd	nd	nd	nd	nd
Granulated onion_a	nd	<LOQ	18.129	4.961	nd
Granulated onion_b	nd	nd	nd	nd	nd
Onion_a	nd	nd	nd	nd	nd
Onion_b	nd	nd	nd	nd	nd
Mix berberè	2491.290	19.099	nd	nd	nd
Spice mix (Curry) _a	nd	12.038	nd	nd	nd
Spice mix (Curry) _b	1712.369	nd	nd	nd	nd
Mix creola	nd	nd	nd	nd	nd
Mix pepper	nd	nd	nd	nd	nd

Table S2: Level (μg/kg) of *Alternaria* mycotoxins in 20 herbs samples without application of the middle-bound approach.

**Herbs (*n*=20)**	(μg/kg)				
	**TeA**	**TEN**	**AOH**	**AME**	**ALT**
Chives	nd	17,476	10,061	nd	nd
Dill	14.899	nd	nd	nd	nd
Sage_a	90.555	nd	57.905	65.377	nd
Sage_b	131.571	nd	17.931	12.852	nd
Thyme	nd	nd	12.439	<LOQ	nd
Oregano_a	101.610	nd	nd	nd	nd
Oregano_b	95.897	13.268	22.532	6.422	nd
Oregano_c	75.718	nd	nd	<LOQ	nd
Basil_a	938.759	23.941	nd	nd	nd
Basil_b	256.222	15.018	nd	nd	nd
Basil_c	nd	nd	nd	nd	nd
Mint	19.706	nd	10.966	<LOQ	nd
Parsley_a	49.186	nd	nd	nd	nd
Parsley_b	nd	nd	nd	nd	nd
Parsley_c	nd	nd	nd	nd	nd
Rosemary_a	31.773	nd	nd	<LOQ	nd
Rosemary_b	nd	nd	nd	nd	nd
Rosemary_c	nd	nd	nd	nd	nd
Marjoram	nd	nd	nd	nd	nd
Mix herbs	51.942	nd	13.470	5.048	nd
